# Artificial intelligence applied to dairy science: insights from the Dairy Brain Initiative

**DOI:** 10.1093/af/vfae040

**Published:** 2025-01-04

**Authors:** Victor E Cabrera

**Affiliations:** Department of Animal and Dairy Sciences, University of Wisconsin–Madison, Madison, WI 53706, United States

**Keywords:** artificial intelligence, dairy brain, dairy science, data analytics, farm management, precision dairy farming

**Implications**

- *Policy Development:* AI-driven data analytics empower policymakers with accurate, real-time information, facilitating more informed decision-making and the creation of policies that promote sustainable agricultural practices.
- *Educational Outreach:* Incorporating AI technologies into agricultural curricula equips the next generation of farmers with the skills needed to navigate the complexities of modern dairy management, ensuring the industry remains innovative and competitive.
- *Sustainability Practices *and E*conomic Advantages**:* By improving resource efficiency and reducing waste, AI contributes significantly to sustainable farming initiatives, helping the dairy industry meet global environmental standards.
  AI technologies also optimize farm operations, leading to significant cost reductions and increased profit margins through improved milk production efficiency and resource management.
- *Animal Health and Welfare:* Advanced AI applications in health monitoring and disease prediction enhance the welfare of dairy herds by providing farmers with tools to anticipate health issues and intervene proactively, improving animal care standards.

## Introduction

The advent of artificial intelligence (AI) has marked a transformative era in dairy science, redefining the approach to data management, farm operations, and animal welfare. Leveraging sophisticated AI technologies, dairy scientists and farmers can now analyze and interpret vast datasets with unprecedented precision and speed. This capacity for deep data analysis enhances decision-making processes, optimizing everything from feeding strategies to disease management and genetic selection. The integration of AI not only bolsters productivity and operational efficiency but also significantly contributes to sustainable farming practices by ensuring optimal resource utilization and reducing environmental impact.

Through the Dairy Brain initiative, this perspective paper explores the cutting-edge applications of AI in dairy science, demonstrating how these technologies are not just augmenting existing practices but fundamentally reshaping the industry. By driving innovations that enhance both the efficiency and sustainability of dairy farms, AI is setting new standards for what is possible in agricultural management.

AI plays a critical role across various domains of dairy science, profoundly impacting how farm operations manage genetic analysis, nutritional planning, and animal health. By integrating AI with existing farm data systems (Wangen et al., 2021), dairy professionals can predict and optimize milk production outcomes through sophisticated algorithms that analyze animal genetics, feed composition, and environmental conditions (Cabrera et al., 2020).

Moreover, AI also extends its utility to disease management by employing predictive models that detect early signs of illness within the herd (Fadul-Pacheco et al., 2021). This capability not only helps in reducing the spread of diseases but also in minimizing treatment costs and downtime for affected livestock. Furthermore, AI supports sustainable practices by ensuring that resource allocation, such as feed and water usage, is managed in the most efficient way possible. This not only conserves resources but also reduces the farm’s overall environmental footprint. Through these diverse applications, AI is revolutionizing dairy science, enabling more precise and informed decision-making that enhances both productivity and sustainability on the farm.

## The Dairy Brain Project

**Figure 1.**
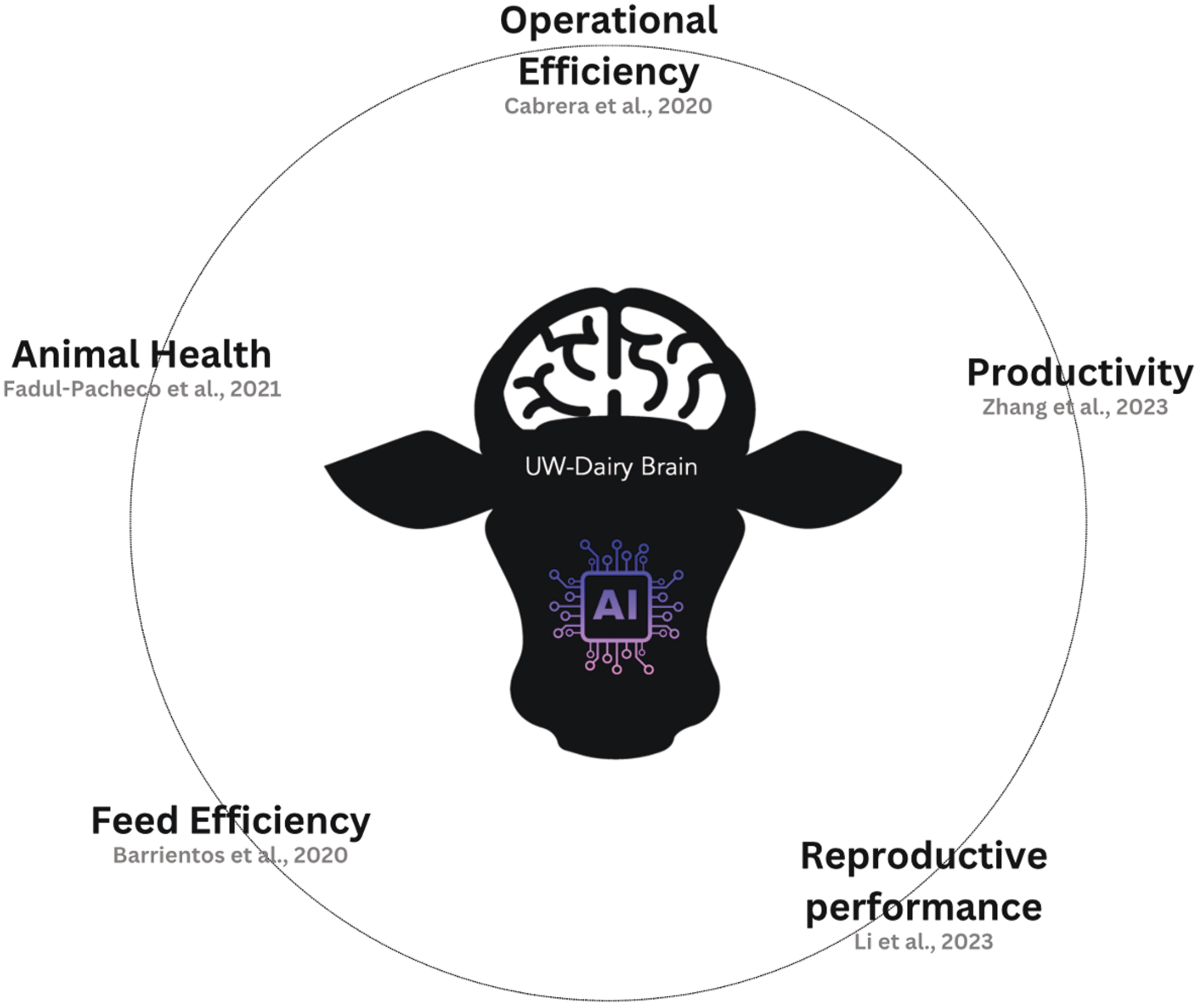
Some Dairy Brain AI insights.

The Dairy Brain project, initiated at the University of Wisconsin–Madison, serves as a pioneering example of how AI can be seamlessly integrated into the operational fabric of dairy farming (Figure 1). This comprehensive system leverages real-time data from a myriad of sources within the dairy operation, including sensors on milking equipment, feed intake monitoring systems, and health tracking devices attached to the animals (Wangen et al., 2021). By collating and analyzing this data through advanced AI algorithms, the Dairy Brain project provides actionable insights that facilitate nuanced decision-making in real time. Cabrera et al. (2020) describe how Dairy Brain facilitates comprehensive data integration, allowing for optimized decision-making that improves both yield and operational efficiency.

Moreover, the Dairy Brain project contributes to environmental sustainability. It optimizes the use of resources by ensuring that feed, water, and energy are utilized efficiently, thereby minimizing waste and reducing the carbon footprint of dairy operations.

Through its multifaceted applications, the Dairy Brain project exemplifies the transformative potential of AI in dairy science. It not only enhances operational efficiency and animal welfare but also supports sustainable practices, showcasing a forward-looking approach to modern dairy management.

## Potential Benefits of AI in Dairy Farming

- *Improved Management:* The integration of AI into various facets of farm operations has led to significant advancements in dairy management. This approach has yielded substantial improvements in operational efficiency, animal health, and economic performance. One notable benefit is the optimization of feed efficiency. By analyzing data related to animal feed and milk output, AI systems determine the most cost-effective feeding strategies without compromising animal health or milk quality (Barrientos et al., 2020). Additionally, predictive analytics are employed to forecast milk yields with greater accuracy (Zhang et al., 2022) and to enhance reproductive management by identifying the most effective breeding protocols, thereby improving overall herd productivity and profitability (Li et al., 2023). AI also plays a crucial role in health management. Continuous monitoring of health data enables early detection of illness or stress in individual animals (Fadul-Pacheco et al., 2021), allowing farm managers to make timely and precise interventions. This proactive approach not only improves animal welfare but also reduces potential losses from disease outbreaks.
- *Operational Efficiency:* The implementation of AI in farm management has revolutionized operations by automating many data-intensive tasks. This automation reduces the burden on farm staff, allowing them to focus on more critical, decision-centric responsibilities. AI’s ability to integrate and analyze data from multiple sources in real time has streamlined the decision-making process, significantly enhancing the efficiency of daily operations. For example, AI-driven systems manage the timing and quantity of feed, optimizing milk production per feed unit and reducing waste. Barrientos et al. (2020) highlighted how optimizing diet accuracy by leveraging data flows already available on the farm not only decreased feed costs by $31 per cow annually but also led to a significant reduction of nitrogen excretion by 5.5 kg per cow per year. These outcomes were achieved by improving the grouping of cows and providing more precise diets, demonstrating both the economic and environmental benefits of data-driven management practices.
- *Animal Health:* By utilizing continuous monitoring systems, AI can detect subtle changes in an animal’s behavior or physiology that may indicate health issues. Utilizing real-time integrated farm data, Fadul-Pacheco et al. (2021) employed machine learning algorithms to predict mastitis cases with 72% accuracy. This result underscores the potential of AI-driven health management systems to enhance early detection and intervention, providing a significant advantage in maintaining herd health and reducing the economic burden associated with disease outbreaks. Early detection allows for timely medical intervention, which can prevent minor ailments from becoming serious, thereby reducing veterinary costs and improving the overall health of the herd. This proactive health management not only supports the welfare of the animals but also ensures consistent milk quality and production.
- *Economic Benefits:* AI-driven solutions have proven to be highly beneficial economically. By enhancing both operational efficiency and animal health, AI helps farmers reduce costs associated with labor, medical interventions, and feed wastage. Moreover, the increased accuracy in predicting optimal breeding times and identifying peak lactation periods maximizes productivity, thereby boosting the profitability of the farm. Li et al. (2023) demonstrated that even small modifications in reproductive management, when guided by integrated data and advanced simulation techniques, could lead to a notable boost in profitability—up to $30 per cow annually. This finding emphasizes the importance of data-informed reproductive strategies in maximizing financial returns on dairy farms, proving that even minor adjustments can yield substantial benefits when supported by robust data analysis. These economic advantages are crucial for the sustainability of dairy farms, particularly in an industry facing narrow margins and fluctuating market conditions.
- *Sustainability:* Beyond immediate farm-level benefits, AI-drive solutions contribute to broader environmental sustainability goals. Efficient resource management, facilitated by AI, leads to reduced waste of feed and water, lower energy consumption, and diminished environmental impact. These sustainable practices not only comply with increasingly stringent environmental regulations but also appeal to consumers who are conscious of the environmental footprint of their food sources. By adopting AI to enhance resource efficiency, farms can align with sustainability standards while also improving their market competitiveness.

In summary, the Dairy Brain project illustrates the transformative potential of AI in enhancing the efficiency, profitability, and sustainability of dairy farms. Its success sets a benchmark for future technological integrations in the agricultural sector, indicating a shift towards more data-driven, responsive, and responsible farming practices (Cabrera and Fadul-Pacheco, 2021).

## Challenges and Considerations in AI Implementation

While AI presents numerous benefits for dairy farming, it is not without challenges that may hinder widespread adoption. One critical issue is *data privacy*. The collection and analysis of large volumes of farm data may raise concerns among farmers about data ownership and how this information is used, particularly when third-party platforms manage the systems. Establishing clear data governance frameworks is essential to ensure farmers retain control over their data and can trust AI systems with sensitive information.

Another challenge is *farmer resistance to technology*. Many farmers, especially those managing small to medium-sized operations, may hesitate to adopt new technologies due to a lack of familiarity or fear that these systems will be too complex or disruptive to their current practices. Education, training, and clear demonstrations of the practical benefits of AI are crucial for overcoming this resistance and encouraging broader acceptance.

Additionally, the *cost of implementation* remains a significant barrier. The investment required for AI systems—including hardware, software, and ongoing maintenance—can be prohibitive for many farms, especially those operating on narrow profit margins. While the long-term benefits of AI may potentially outweigh these initial costs, financial incentives, subsidies, or cost-sharing models could be necessary to make these technologies accessible to a wider range of farms.

*Scalability* also presents a challenge, particularly for small or limited-resource farms. Implementing AI systems requires substantial investment in technology, infrastructure, training, and ongoing maintenance, which can be difficult for operations with fewer financial or technical resources. Many farms may also lack the digital infrastructure or expertise needed to fully integrate AI into their management systems. As a result, these farms may struggle to scale AI solutions to meet their specific needs and constraints. Nonetheless, once the initial challenges of technology adoption and system integration are addressed, AI systems hold significant potential for scalability. This would allow broader adoption and greater flexibility in application across farms with diverse operational structures, providing tailored, data-driven solutions that enhance efficiency and productivity, even for operations with limited resources.

Beyond these key challenges, additional barriers may affect the adoption of AI in dairy farming, such as the need for reliable internet infrastructure, the complexity of integrating AI systems with existing farm technologies, and concerns about the long-term maintenance of AI tools. Addressing these challenges is essential to ensure that AI can reach its full potential in transforming dairy farming. Highlighting these areas also points to where further research and support are needed to facilitate successful AI adoption.

## Future Directions in AI for Dairy Science

As AI continues to evolve rapidly, its applications in dairy science are expected to broaden and deepen, bringing even more sophisticated tools to the forefront of agricultural technology. Future research is likely to focus on enhancing the capabilities of AI to provide even more precise analytics for genetic selection, disease prediction, and environmental management. Innovations may include the integration of AI with genomic information to accelerate breeding programs that prioritize health, efficiency, and resilience in dairy herds.

Additionally, AI is set to play a crucial role in advancing sustainable practices within the industry. This includes the development of systems that can more accurately monitor and manage the carbon footprint of dairy operations (e.g., *DairyPrint model*; da Silva and Cabrera, 2024). New algorithms are also expected to improve resource allocation, such as water and feed, reducing waste and increasing efficiency. As these technologies become more accessible, they promise to democratize high-level dairy management tools, making them available to farms of all sizes, thereby enhancing the overall productivity and sustainability of the global dairy industry.

However, addressing challenges related to trustworthiness, scalability, and ethical considerations will be crucial for the successful integration of AI into dairy farm operations. By fostering collaboration between researchers, industry partners, and policymakers, the dairy industry can harness the full potential of AI while ensuring responsible and sustainable practices.

## Conclusion

AI is reshaping dairy science, bringing about a new era of efficiency and precision in farm management. The Dairy Brain project illustrates the significant benefits of AI adoption, highlighting improvements in operational efficiency, animal welfare, and economic performance. As the industry continues to evolve, AI will play an increasingly central role in meeting the challenges of modern dairy farming, making it an indispensable tool in the quest for sustainability and profitability.

## About the Author

Dr. Victor E. Cabrera is a professor at the University of Wisconsin–Madison, where he combines his expertise in dairy science with applied economics and cutting-edge technology. A pioneer in the application of AI to dairy management, Dr. Cabrera’s work, particularly on the Dairy Brain project, has significantly influenced global dairy farming practices. Dr. Cabrera has authored numerous influential publications that effectively bridge the gap between complex data analytics and practical applications, making substantial contributions to the field.

**Corresponding author:** vcabrera@wisc.edu
